# DHA Supplementation of Obese Rats throughout Pregnancy and Lactation Modifies Milk Composition and Anxiety Behavior of Offspring

**DOI:** 10.3390/nu13124243

**Published:** 2021-11-26

**Authors:** Elena Zambrano, Guadalupe L. Rodríguez-González, Luis A. Reyes-Castro, Claudia J. Bautista, Diana C. Castro-Rodríguez, Gimena Juárez-Pilares, Carlos A. Ibáñez, Alejandra Hernández-Rojas, Peter W. Nathanielsz, Sara Montaño, Armando Arredondo, Fengyang Huang, Francisco Bolaños-Jiménez

**Affiliations:** 1 Reproductive Biology Department, Instituto Nacional de Ciencias Médicas y Nutrición Salvador Zubirán, Mexico City 14080, Mexico; guadalupe.rodriguezg@incmnsz.mx (G.L.R.-G.); lafe_mat@hotmail.com (L.A.R.-C.); claudia.bautistac@incmnsz.mx (C.J.B.); diana.castror@incmnsz.mx (D.C.C.-R.); daycna@hotmail.com (G.J.-P.); carlos_albertoibc@hotmail.com (C.A.I.); alehero03@gmail.com (A.H.-R.); 2 CONACyT-Cátedras, Reproductive Biology Department, Instituto Nacional de Ciencias Médicas y Nutrición Salvador Zubirán, Mexico City 14080, Mexico; 3 Department of Animal Science, University of Wyoming, Laramie, WY 82071, USA; peter.nathanielsz@uwyo.edu; 4 Department of Animal Nutrition, Instituto Nacional de Ciencias Médicas y Nutrición Salvador Zubirán, Mexico City 14080, Mexico; sara.montanob@incmnsz.mx; 5 Center for Health Systems Research, Instituto Nacional de Salud Pública, Cuernavaca 62100, Mexico; armando.arredondo@insp.mx; 6 Laboratory of Pharmacology and Toxicology, Hospital Infantil de México Federico Gómez, Mexico City 06720, Mexico; f_y_huang@yahoo.com; 7 INRAE, UMR1280 Physiologie des Adaptations Nutritionnelles, Université de Nantes, Nantes Atlantique Université, 44096 Nantes, France; francisco.bolanos@univ-nantes.fr

**Keywords:** pregnancy, obesity, breastfeeding, behavioral disorders, LCPUFA, interventions

## Abstract

We investigated if supplementing obese mothers (MO) with docosahexaenoic acid (DHA) improves milk long-chain polyunsaturated fatty acid (LCPUFA) composition and offspring anxiety behavior. From weaning throughout pregnancy and lactation, female Wistar rats ate chow (C) or a high-fat diet (MO). One month before mating and through lactation, half the mothers received 400 mg DHA kg^−1^ d^−1^ orally (C+DHA or MO+DHA). Offspring ate C after weaning. Maternal weight, total body fat, milk hormones, and milk nutrient composition were determined. Pups’ milk nutrient intake was evaluated, and behavioral anxiety tests were conducted. MO exhibited increased weight and total fat, and higher milk corticosterone, leptin, linoleic, and arachidonic acid (AA) concentrations, and less DHA content. MO male and female offspring had higher ω-6/ ω-3 milk consumption ratios. In the elevated plus maze, female but not male MO offspring exhibited more anxiety. MO+DHA mothers exhibited lower weight, total fat, milk leptin, and AA concentrations, and enhanced milk DHA. MO+DHA offspring had a lower ω-6/ω-3 milk intake ratio and reduced anxiety vs. MO. DHA content was greater in C+DHA milk vs. C. Supplementing MO mothers with DHA improves milk composition, especially LCPUFA content and ω-6/ω-3 ratio reducing offspring anxiety in a sex-dependent manner.

## 1. Introduction

Obesity in children and women of reproductive age is a worldwide health problem that has reached epidemic proportions [1,2,3]. Maternal obesity during pregnancy has a negative impact on the health of both mothers and their babies [4,5,6]. Obese pregnant women, for example, are more likely to develop gestational diabetes, hypertension, and pre-eclampsia [7,8], as well as being less likely to initiate breastfeeding [9,10,11], and when they do, they breastfeed for a shorter period of time, reducing the offsprings’ benefits of breastfeeding [12].

According to epidemiological studies, breastfeeding protects infants from rapid neonatal weight gain and obesity susceptibility later in life [13] and plays a key role in preventing childhood behavioral disorders [14]. However, recent evidence suggests that the extent of breastmilk’s benefits are modulated by its fatty acid composition [15], such as long-chain polyunsaturated fatty acids (LCPUFA). LCPUFAs such as eicosapentaenoic acid (EPA), docosahexaenoic acid (DHA), and arachidonic acid (AA) play important roles during lactation as they are key constituents of cell membranes, the nervous system, and the retina in the infant [16,17].

Infants’ ability to synthesize LCPUFA, particularly DHA, is reduced. As a result, for neonates, breastmilk is by far the greatest source of these fatty acids [18]. In previous studies, we have demonstrated that maternal obesity induced by a high-fat diet has an effect on maternal milk nutrient concentrations, including an increase in milk fat content, a decrease in DHA and EPA, and an increase in AA. In later life, these changes predispose offspring to greater fat accumulation, metabolic problems, cognitive alterations, and increased anxiety-like behavior [19,20]. These poor offspring outcomes have been associated with the altered availability of LCPUFA during fetal development and/or lactation. [15,21,22,23]. In addition, regulatory compounds in milk such as cortisol or leptin can potentially influence offsprings’ behavior and growth trajectory [24,25]. It has been shown that some of the negative outcomes linked with low LCPUFA availability during development are mitigated by maternal ω-3 supplementation during pregnancy [26,27,28]. However, in the context of developmental programming, there have been a limited number of studies on the effects of DHA supplementation before and during pregnancy and lactation in obese rat pregnancies and its impact on milk composition and offspring behavior. We hypothesized that DHA supplementation in obese pregnant rats would improve the adverse changes in maternal milk LCPUFA concentration and offspring anxiety behavior.

## 2. Methods

### 2.1. Females Recruited for Breading as Mothers for Offspring Study Production

The Animal Experimentation Ethics Committee of the Instituto Nacional de Ciencias Médicas y Nutrición Salvador Zubirán (INCMNSZ), Mexico City, Mexico (ethical approval code, BRE-1870) approved all procedures, which are in accordance with the ARRIVE criteria for reporting animal studies [29,30]. Female albino Wistar rats were born and raised in the INCMNSZ animal facility, which is accredited by the Association for Assessment and Accreditation of Laboratory Animal Care International (AAALAC) and follows its criteria. Rats were maintained at a constant temperature (22–23 °C) and under controlled lighting (lights on 07:00 to 19:00 h) and fed standard laboratory chow diet (LabRodent Diet 5001, Fort Worth, TX, USA) containing 23.9% protein, 5.0% fat, 31.9% polysaccharide, 6.2% simple sugars, 5.0% fiber, 7.0% minerals and ~1.0% vitamins (*w*/*w*), physiological fuel 3.4 kcal g^−1^ (29% as protein, 58% as CHO, 13% as fat). At 14–16 weeks of age (weighing 200–240 g), females were randomly assigned to breed with non-litter mates of proven fertility. At delivery (day 0), litters that provided Founder Generation (F0) mothers were culled to 10 pups, each with at least four females. At weaning (day 21), one female F0 pup from each litter was randomly assigned to either a maternal control (C, *n* = 16) group fed a conventional laboratory chow diet or a maternal obesity (MO, *n* = 14) group fed a high-fat diet [31] containing 23.5% protein, 20.0% lard, 5.0% corn oil fat, 20.2% polysaccharide, 20.2% simple sugars, 5.0% fiber, 5.0% mineral mix, 1.0% vitamin mix (*w*/*w*), physiological fuel 4.8 kcal g^−1^ (17% as protein, 35% as CHO, 47% as fat). The high-fat diet was developed at the INCMNSZ’s specialized dietary unit. To ensure homogeneity in the developmental programming challenge and maternal genetics to which offspring were exposed by F0 mothers, only one F0 female from the same litter was included in any experimental group.

At postnatal day (PND) 90, 1 month before mating and during pregnancy and lactation, half of the F0 females from each group (C and MO) were maintained on their assigned diet and received 400 mg DHA · kg^−1^ d^−1^ (DSM Nutritional Products, Inc., Heerlen, Netherlands) daily orally as an individual dose by pipette to generate 2 additional groups, C+DHA and MO+DHA. On PND 120, F0 female rats were mated with proven male breeders and conceived the next cycle. Lactating mothers were kept on their prenatal diets (C or MO) and with or without DHA supplementation (C+DHA and MO+DHA).

### 2.2. Measurement of Milk Composition

Milk composition studies must always pay attention to collection timing and validation of assay techniques [32]. Milk was obtained between 11:00 and 13:00 h on day 21 of lactation (dL). Pups were removed from mothers after 4 h, and mothers were given 0.8 U of oxytocin (ip) and milked 15 min later, as previously reported. [33]. Milk samples were vortexed to mix them completely, then separated into aliquots and stored at −20 °C until they were analyzed. To achieve sample homogeneity, milk samples were thawed at 37 °C and vigorously shaken. Gravimetric analysis was used to determine the water concentration (%) [34]. Protein concentration (%) was determined using the Bradford test (Biorad^®^, Hercules, CA, USA). Total fat concentration (%) was determined using the Folch technique [35]. The concentrations of linoleic acid, AA, EPA, and DHA in milk fat were determined using gas chromatography [33].

### 2.3. Milk Production and Pup Intake of Different Milk Components

Milk production was estimated based on detailed descriptions provided by us and other investigators [36,37,38]. Briefly, at 20 dL and at 07:00 h, pups were removed for 4 h from their mothers. Only dams had access to water and food ad libitum (to produce milk). Mothers were weighed at the beginning and end of the 4 h period. Pups were individually weighed before being returned to their mothers and then again 1 h later. Approximations of male and female pup milk component intake (water, protein, fat, linoleic acid, AA, EPA, and DHA) were estimated by milk intake (g h^−1^) × milk component (%) 100^−1^.

### 2.4. Fatty Acid Analysis

Milk lipids were extracted using a modified Folch technique. Samples were homogenized with 2 mL of 0.9% NaCl and 5 mL of chloroform:methanol (2:1) as previously described [35,39]. Fatty acid extraction was carried out using chloroform (3 × 2 mL). The organic phase was pooled, and 120–150 µL of methanol was added until the organic phase turned transparent, then 1 g of Na_2_SO_4_ was added and vortexed to obtain the residue for fatty acid analysis. A stream of nitrogen was used to evaporate the organic phase.

### 2.5. Preparation of Fatty Acid Methyl Esters

Samples of fatty acid methyl esters (FAME) were prepared as previously described [39]. Briefly, FAMEs were extracted using hexane, and the organic phase was pooled and evaporated under a stream of nitrogen. Hexane was added to the residue and centrifuged before being injected into a gas chromatograph with a flame ionization detector model 6850 (Agilent, Santa Clara, CA, USA). The retention times for methyl ester standards (PolyScience, Niles, IL, USA) we used to identify fatty acid methyl esters, and each one was reported as a percentage of total fatty acid in the sample.

### 2.6. Blood Collection and Hormone Quantification

On day 21 (end of lactation), F0 rats from all groups were weighed and euthanized under general anesthesia with isoflurane, followed by decapitation using a rodent guillotine (Thomas Scientific, Swedesboro, NJ, USA) by trained staff knowledgeable in the technique. Blood was drawn from the trunk, and serum was separated and stored at −70 °C. Corticosterone and leptin concentrations in both serum and milk were determined by enzyme-linked immunosorbent assay (ELISA), using commercial rat kits from DRG International, Inc. (Springfield Township, NJ, USA) and Invitrogen (Carlsbad, CA, USA), respectively. Each sample was measured in duplicate. The % of fat (Folch) and the adiposity index (AI) were determined by excising and weighing the mammary gland and fat depots, respectively. AI = total adipose tissue × 100 · body weight g^−1^. We report here data with the following number of mothers, and offspring of each sex—C: *n* = 8, C+DHA: *n* = 8, MO: *n* = 7, MO+DHA: *n* = 7.

### 2.7. Offspring (F1) Maintenance

To guarantee F1 homogeneity, on postnatal day (PND) 2, all F0 litters from all groups studied were adjusted to 10 pups, with equal numbers of males and females wherever possible. Litters with fewer than 11 or more than 14 pups were excluded from the study. At weaning (PND 21), F1 litters were divided into male and female offspring and housed 5 per cage and fed standard laboratory chow diet throughout the study. No litters or sexes from different treatment or age groups were mixed together. After PND 50, a maximum of 3 rats of the same sex and experimental group were housed per cage. On PND 110, behavioral tests were conducted on male and female F1.

### 2.8. Behavioral Assessment

#### 2.8.1. Elevated Plus Maze (EPM)

Two weeks prior to behavioral testing, a reverse light cycle was implemented with lights turned off at 07.00 h and on at 19.00 h. At PND 110, F1 rats were evaluated during the dark phase (between 08.00 h and 14.00 h). The EPM’s specifications have been described in detail [40]. Briefly, rats were placed in the EPM and allowed to explore for 5 min. The Ethovision system (Ethovision, Noldus Information Technology by Wageningen, The Netherlands) kept track of the number of entries, distance traveled, and time spent in open and closed arms. The number of false entries into the different areas was manually scored by an experimenter who was blind to the subject’s treatment group. A false entry was considered when less than 50% of the rat’s body was not inside the arms, whereas an arm entry only was scored if the rat’s center of gravity entirely entered the arm. False entries were subtracted from the total number of entries that were automatically counted. All subjects were tested in a randomized sequence. Females’ behavioral assessments were performed during diestrous.

#### 2.8.2. Open Field (OF)

The day after EPM testing, the same subjects were evaluated in a 10-min OF test. The specifications of the OF have been described in detail [40]. Briefly, the Ethovision system was used to measure overall distance (measured in meters), number of entries and time spent, and distance traveled inside the center zone.

### 2.9. Statistical Analysis

All data are presented as mean ± SEM, *n* = 7–8 per group from different litters. Body weight, a basic and well-characterized biologically meaningful endpoint, was examined by one-sided t-test in male and female F1 from the control group and was statistically different (*p* < 0.001), therefore sexes were analyzed separately. To assess the statistical differences within maternal diet and DHA supplementation groups, data were analyzed using two-way multiple analysis of variance (ANOVA), followed by Tukey test. Corticosterone and leptin concentrations in milk were compared to serum, and Pearson’s correlations were calculated. *p* ≤ 0.05 was considered significant. Log-transformation was used for skewed and spread data (serum and milk corticosterone, and milk leptin).

## 3. Results

### 3.1. Maternal Food Intake during Lactation

**Figure 1:**
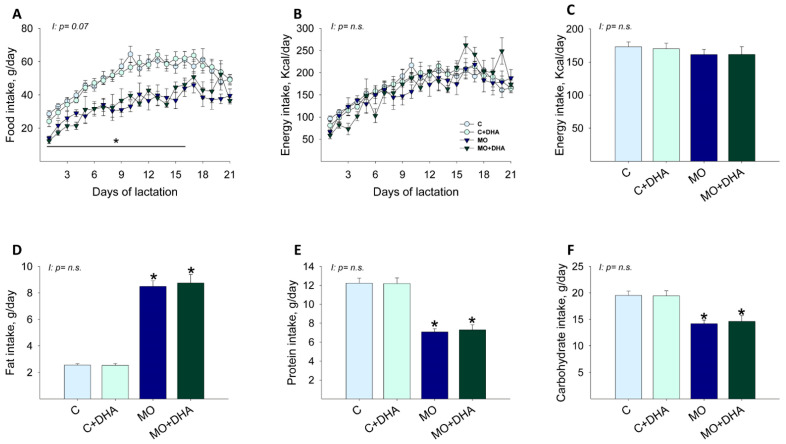
Maternal food, energy, and macronutrient intake during lactation. Control (C), control with DHA (C+DHA), maternal obesity (MO), and maternal obesity with DHA (MO+DHA). (**A**). food intake, g/day; (**B**). energy intake, kcal/day; (**C**). average energy intake during lactation, kcal/day; (**D**). fat intake, g/day; (**E**). protein intake, g/day; (**F**). carbohydrate intake, g/day. Values are mean ± SEM, *n* = 7–8 mothers per group. * different vs. their respective control, *p* < 0.05. I = interaction between maternal diet and maternal DHA supplementation, n.s. = not significant.

From lactation day 1–16, maternal food intake in g/day was reduced in MO and MO+DHA groups compared with C and C+DHA, respectively (Figure 1A). Maternal energy intake per day, as well as average energy intake during lactation, were similar in all groups (Figure 1B,C). Maternal fat intake was higher, while protein and carbohydrate intake was lower in MO and MO+DHA groups in comparison with C and C+DHA (Figure 1D–F).

### 3.2. Maternal Parameters at the End of Lactation

**Figure 2:**
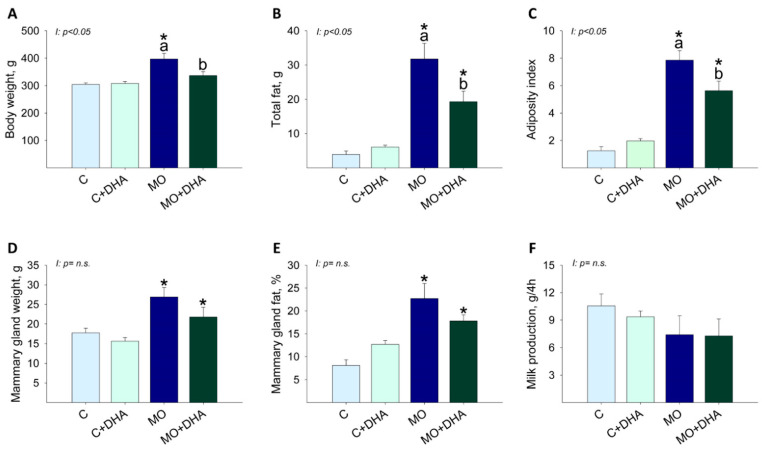
Maternal parameters at the end of lactation. Control (C), control with DHA (C+DHA), maternal obesity (MO), and maternal obesity with DHA (MO+DHA). (**A**). body weight, g; (**B**). total fat, g; (**C**). adiposity index; (**D**). mammary gland weight, g; (**E**). fat in mammary gland, %; (**F**). milk production, g/4h. Values are mean ± SEM, *n* = 7–8 mothers per group. Within the same group, means labeled with different letters differ, *p* < 0.05. * different vs. their respective control, *p* < 0.05. I = interaction between maternal diet and maternal DHA supplementation, n.s. = not significant.

Body weight was higher in MO mothers compared with C, while maternal DHA supplementation reduced MO+DHA body weight as compared with MO (Figure 2A). MO and MO+DHA had higher total fat and adiposity index than C and C+DHA, respectively; maternal DHA supplementation reduced total fat and adiposity index in MO+DHA compared to MO (Figure 2B,C). Mammary gland weight was higher in mothers from MO and MO+DHA groups compared with C and C+DHA (Figure 2D); mammary gland fat concentration was higher in MO and MO+DHA as compared with C and C+DHA, respectively (Figure 2E). Milk production was similar across all groups (Figure 2F).

### 3.3. Maternal Hormonal Concentrations at the End of Lactation

**Figure 3:**
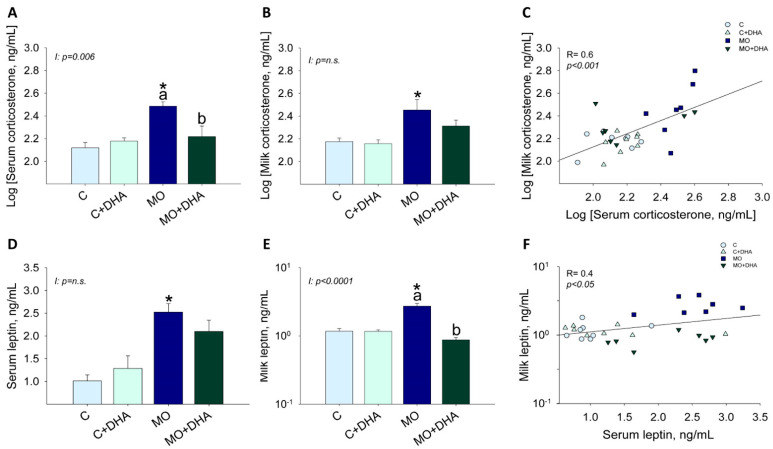
Maternal hormone concentrations at the end of lactation. Control (C), control with DHA (C+DHA), maternal obesity (MO), and maternal obesity with DHA (MO+DHA). (**A**). Log [serum corticosterone], ng/mL; (**B**). Log [milk corticosterone], ng/mL; (**C**). Log [milk corticosterone], ng/mL vs. Log [serum corticosterone], ng/mL correlation; (**D**). serum leptin, ng/mL; (**E**). milk leptin, ng/mL in log scale; (**F**). milk leptin, ng/mL in log scale vs. serum leptin, ng/mL correlation. Values are mean ± SEM, *n* = 7–8 mothers per group. Within the same group, means labeled with different letters differ, *p* < 0.05. * different vs. their respective control, *p* < 0.05. I = interaction between maternal diet and maternal DHA supplementation, n.s. = not significant. R = Pearson’s correlation.

Maternal corticosterone and leptin concentrations in both serum and milk were increased in MO compared with C (Figure 3A,B,D,E); maternal DHA supplementation reduced serum corticosterone and milk leptin concentrations in MO+DHA to levels comparable with C and C+DHA (Figure 3A,E). Corticosterone concentrations in serum and milk showed a highly significant positive correlation (R = 0.6, *p <* 0.001) (Figure 3C); there was also a positive correlation between leptin concentrations in serum and milk (R = 0.4, *p* < 0.05) (Figure 3F).

### 3.4. Milk Nutrient Composition at the End of Lactation

**Figure 4:**
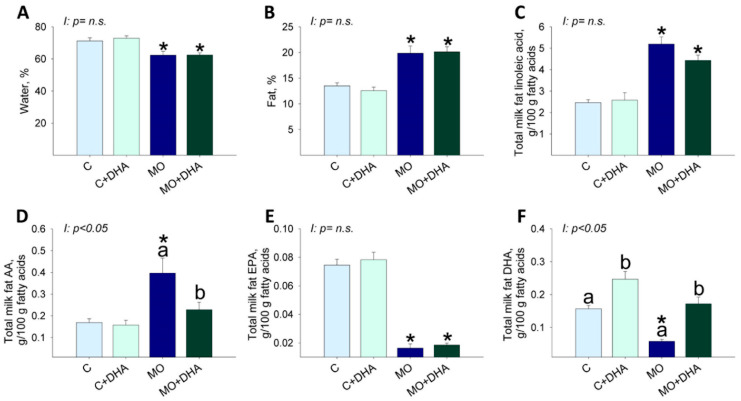
Maternal milk composition at the end of lactation. Control (C), control with DHA (C+DHA), maternal obesity (MO), and maternal obesity with DHA (MO+DHA). (**A**). water, %; (**B**). fat, %; (**C**). linoleic acid, %; (**D**). AA, %; (**E**). EPA, %; (**F**). DHA, %. Values are mean ± SEM, *n* = 7–8 rats from different litters. Within the same group, means labeled with different letters differ, *p* < 0.05. * different vs. their respective control, *p* < 0.05. I = interaction between maternal diet and maternal DHA supplementation, n.s. = not significant.

MO and MO+DHA milk had lower percentages of water concentrations than C and C+DHA (Figure 4A), but protein levels were similar among groups (C: 6.5 ± 0.5, C+DHA: 4.7 ± 0.8, MO: 6.5 ± 0.4, MO+DHA: 6.6 ± 0.5). The percentage of fat concentration in milk was higher in MO and MO+DHA compared with C and C+DHA, respectively (Figure 4B). Milk linoleic acid and AA concentrations were higher in MO than in C, while maternal DHA supplementation only reduced AA in MO+DHA (Figure 4C,D). Milk EPA concentration was lower in both MO and MO+DHA compared with C and C+DHA (Figure 4E). MO milk had a lower DHA concentration than C (Figure 4F). Maternal DHA supplementation increased milk DHA concentration in both C+DHA and MO+DHA groups when compared to C and MO, respectively (Figure 4F). The percentage of ω-3 PUFA was similar among groups. The percentage of ω-6 PUFA was higher in both MO and MO+DHA compared with C and C+DHA, respectively. The monosaturated acids (MSA) were only higher in MO+DHA in comparison with C+DHA, and the saturated fatty acids (SFA) were only higher in MO than in C (Table 1).

### 3.5. Pup Nutrient Intake in Milk at the End of Lactation

**Figure 5:**
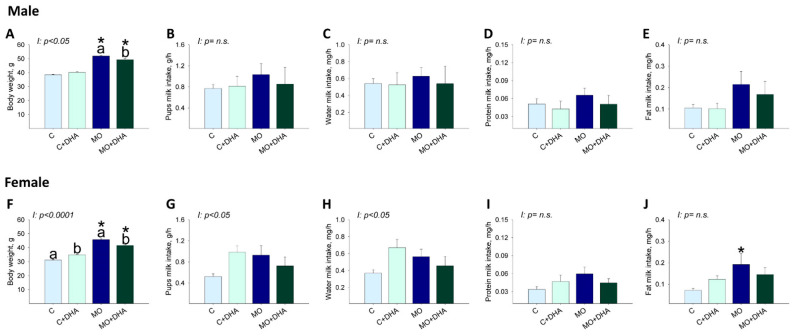
Male and female pups milk intake at the end of lactation. Control (C), control with DHA (C+DHA), maternal obesity (MO), and maternal obesity with DHA (MO+DHA). Male and female: (**A**,**F**). body weight, g; (**B**,**G**). milk intake, g/h; (**C**,**H**). water intake, mg/h; (**D**,**I**). protein intake, mg/h; (**E**,**J**). fat intake, mg/h. Values are mean ± SEM, *n* = 7–8 rats from different litters. Within the same group, means labeled with different letters differ, *p* < 0.05. * different vs. their respective control, *p* < 0.05. I = interaction between maternal diet and maternal DHA supplementation, n.s. = not significant.

At the end of lactation, male and female pups’ body weight was higher in MO and MO+DHA than in C and C+DHA, respectively (Figure 5A,F). Maternal DHA supplementation reduced male and female pups’ body weight in MO+DHA compared to MO group; C+DHA female pups weighed more than C (Figure 5F). Across all groups, male pups consumed similar amounts of milk, water, protein, and fat (Figure 5B–E).

Milk consumption, as well as water and protein milk intake in female pups, was similar across groups (Figure 5G–I). However, fat milk intake was higher in MO females than in C (Figure 5J).

**Figure 6:**
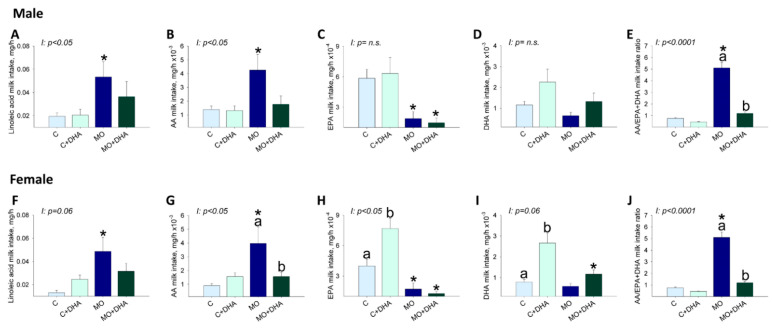
Pups’ milk intake at the end of lactation. Control (C), control with DHA (C+DHA), maternal obesity (MO), and maternal obesity with DHA (MO+DHA). Male and female: (**A**,**F**). linoleic acid intake, mg/h; (**B**,**G**). AA intake, mg/h; (**C**,**H**). EPA intake, mg/h, (**D**,**I**). DHA intake, mg/h (**E**,**J**). AA/EPA+DHA intake ratio. Values are mean ± SEM, *n* = 7–8 rats from different litters. Within the same group, means labeled with different letters differ, *p* < 0.05. * different vs. their respective control, *p* < 0.05. I = interaction between maternal diet and maternal DHA supplementation, n.s. = not significant.

Milk linoleic acid intake was higher in male and female offspring from the MO group compared with C (Figure 6A,F), and maternal DHA supplementation did not reduce male (*p* = 0.09) or female (*p* = 0.07) milk linoleic acid intake. Milk AA intake was higher in male and female MO offspring compared to C, whereas maternal DHA supplementation reduced AA intake in female but not in male pups (*p* = 0.05) from the MO+DHA group compared to MO (Figure 6B,G). EPA intake was lower in both male and female pups from MO and MO+DHA compared to C and C+DHA (Figure 6C,H) but higher in female offspring from C+DHA vs. C (Figure 6H). Milk DHA intake was significantly higher in females from C+DHA group compared with C, while in MO + DHA females, DHA intake was lower than in C+DHA (Figure 6I); in males, DHA intake was similar among groups (Figure 6D). The AA/EPA+DHA milk intake ratio was similar in the C and C+DHA groups but higher in male and female MO offspring compared to C. The ratio of AA/EPA+DHA milk intake in MO+DHA offspring was reduced by maternal DHA supplementation (Figure 6E,J). Male and female ω-6 PUFA intake was higher in MO than in C. SFA intake was higher in female pups from MO compared to C, but unchanged in male pups. ω-3 PUFA and MSA milk intake were similar in males and females among all experimental groups (Table 2). Male and female pups from MO and MO+DHA groups had higher ω-6/ω-3 fatty acid intake ratios than C and C+DHA, respectively. Maternal DHA supplementation reduced the ω-6/ω-3 fatty acid intake ratios in C vs. C+DHA and MO+DHA vs. MO (Table 2).

### 3.6. Elevated Plus Maze Studies

**Figure 7:**
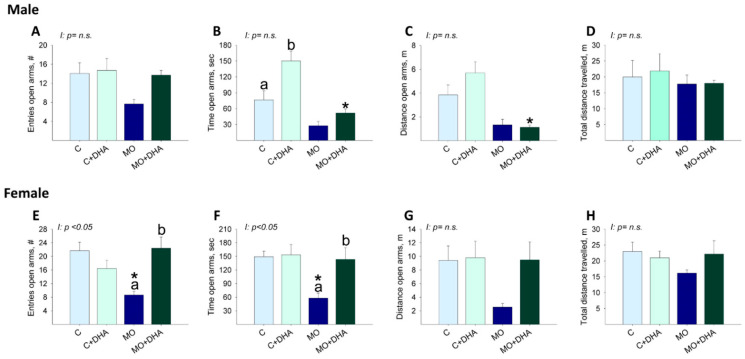
Elevated plus maze in offspring at PND 110. Control (C), control with DHA (C+DHA), maternal obesity (MO), and maternal obesity with DHA (MO+DHA). Male and female: (**A**,**E**). open arm entries, #; (**B**,**F**). open arm time, sec; (**C**,**G**). open arm distance, m; (**D**,**H**). total distance travelled, m. Values are mean ± SEM, *n* = 7–8 rats from different litters. Within the same group, means labeled with different letters differ, *p* < 0.05. * different vs. their respective control, *p* < 0.05. Interaction between maternal diet and maternal DHA supplementation, n.s. = not significant.

Male offspring behavior: the number of open arm entries was similar in F1 across groups (Figure 7A). C+DHA pups spent more time in the open arms than C, whereas MO+DHA offspring spent the least time in the open arms compared with C+DHA (Figure 7B). In MO+DHA, the distance traveled in the open arms was lower than in C+DHA (Figure 7C). The total distance traveled was similar across all groups (Figure 7D).

Female offspring behavior: MO F1 had fewer entries and spent less time in the open arms than C. Maternal DHA supplementation increased in MO+DHA the open arm entries, as well as time spent in the open arms compared with MO (Figure 7E,F). No differences were observed between groups in the distance traveled in the open arms or in the total distance traveled (Figure 7G,H).

### 3.7. Open field Studies

Male offspring behavior: MO center zone entries, as well as time spent in the center zone, was lower compared with C. Center zone distance as well, as total distance traveled was similar among groups (Table 3).

Female offspring behavior: Center zone entries, time and distance, as well as total distance traveled, were similar among groups (Table 3).

## 4. Discussion

Obesity rates in women of reproductive age have increased dramatically. Children of obese mothers are more susceptible to metabolic and heart diseases, as well as behavioral problems across their lifespan [41]. As a result, there is an urgent need to develop strategies to control the negative effects of maternal obesity on offsprings’ health. Some studies have addressed pregnancy and lactation as critical time windows for early prevention of negative offspring health outcomes programmed by MO [19,20,42,43]. The long-chain ω-3 PUFAs, EPA, and DHA are essential nutritional components, exerting important anti-inflammatory, hypolipidemic, and neurological effects, especially during development [44,45]. However, few experimental studies have investigated the potential benefits of maternal DHA supplementation in obese rat mothers before, during, and after pregnancy and lactation on milk composition and offspring anxiety-like behavior. We hypothesized that DHA supplementation in obese pregnant rats improves milk LCPUFA concentration and offspring anxiety behavior.

Dysregulation of energy homeostasis due to a disproportionate consumption of diets high in fat, calories, or sugars is a major cause of excessive weight gain, as well as decreased ω-3 PUFA intake [46]. In previous studies [20,33,43], we have shown that a high-fat diet consumption prior to and throughout pregnancy and lactation increases maternal body weight and total body fat in MO mothers at the end of lactation. The observation of the same maternal phenotype in the current study demonstrates the robustness and reproducibility of our model.

Consumption of a high-fat diet is considered one of the major causes of obesity since high-fat diets are generally high in energy. In our model, during the lactation period, MO mothers exhibited a reduction in food intake without changes in energy intake; this can be explained by the fact that since their own weaning, MO mothers have been fed with the high-fat diet, resulting in a decreased appetite [47]. The chow diet was used as a control diet because our group [48] and others [49] have found that the use of purified diets raises serum triglyceride concentrations.

Animal studies suggest that increased consumption of ω-3 can reduce body fat in rodents fed a high-fat diet [50,51], possibly as a result of altered gene expression and protein function modulation of metabolic pathways related to energy balance, endocrine changes, and inflammation [52]. Leptin is secreted in proportion to body fat mass by adipocytes and plays an important role in energy homeostasis by acting as a negative feedback regulator of appetite in the brain. Thus, decreased leptin signaling promotes positive energy balance and fat accumulation. MO increases maternal leptin serum concentrations, which is explained by the increased body weight, total fat, and adiposity index [53]. In the present study, maternal DHA supplementation in obese rats (MO+DHA) decreased maternal body weight, total fat, adiposity index, and mammary gland weight. These findings suggest that DHA alters the balance of lipogenesis and lipolysis. Animal studies suggest that early PUFA exposure influences adipose tissue development because AA promotes adipocyte maturation while DHA inhibits this process. Offspring of rats fed a diet rich in ω-3 PUFAs during pregnancy and lactation had lower body weight, adipose tissue mass, and leptin concentrations than offspring of mothers fed a diet low in ω-3 PUFAs [54]. These differences could explain the increase in pup body weight in the MO group and the decrease in pup body weight due to maternal DHA supplementation.

Breast milk is the first food consumed by mammals and contains all of the energy and nutrients required for normal offspring growth and development [25]. Breast milk also contains proteins, minerals, vitamins, fats, sugars, antibodies, and growth factors [55], as well as biologically active hormones such as glucocorticoids and leptin [56,57]. Glucocorticoids influence metabolic processes such as carbohydrate, protein, and fat metabolism, as well as cognition, behavior, and stress responses [56]. Furthermore, glucocorticoids play an important role in milk secretion by inducing mammary gland development of ultrastructural components (rough endoplasmic reticulum and tight junctions), regulating milk protein gene expression, and controlling enzymes that are responsible for lipogenesis, lactose synthesis, and the viability and function of mammary secretory cells [58]. There is no firm evidence for or against the synthesis of glucocorticoids by the mammary gland. However, evidence indicates that milk glucocorticoids are transferred directly from plasma to breast milk [59]. The infant’s intestinal tract contains a high number of glucocorticoid receptors in early life. Milk glucocorticoids that reach the infants’ intestines can thus easily cross the intestinal epithelial barrier and then the blood-brain barrier [60].

Our results demonstrate that maternal high-fat diet consumption increased corticosterone concentrations in both serum and milk and that maternal DHA supplementation in MO+DHA partially reduced corticosterone concentrations to levels comparable to C and MO. It has been reported that plasma and breast milk glucocorticoids are correlated [61]. In line with this finding, our results showed a positive correlation between serum and milk corticosterone. In control pregnant mice, it has been reported that supplementing the diet with DHA and AA reduced corticosterone serum concentrations in the offspring at adulthood [62]. In a study using isolated porcine microsomes, it has been suggested that ω-3 (DHA) impedes adrenal glucocorticoid production [63]. In addition, chronic dietary ω-3 PUFA supplementation has been shown to prevent chronic stress-induced emotional and neuronal impairment by inhibiting hypothalamic–pituitary–adrenal (HPA) axis hyperactivity [64]. However, the mechanisms underlying these effects on glucocorticoid function are not well understood.

Leptin, an adipocyte-derived hormone, is synthesized by the mammary gland [65]. Leptin is also transferred from maternal blood to breast milk and from the milk to the pup’s blood [66]. In rodents, between days 5 and 10 of lactation, milk and maternal plasma leptin concentrations correlate positively. However, by late lactation, maternal milk leptin concentrations are independent of maternal plasma leptin concentrations [65]. In the present study, we observed that at the end of lactation, leptin concentrations in both serum and milk were increased in MO mothers. Interestingly maternal DHA supplementation had no effect on serum leptin but did reduce milk leptin concentration. Maternal DHA supplementation has a direct effect on mammary gland growth and maturation as well as transport functions during lactation. Supporting this view, ω-3 supplementation in non-lactating mice modulates mammary gland structure, fatty acid composition, and inflammatory processes [67]. There is a need for further studies into the effects of maternal DHA supplementation in obese rats’ mammary gland development and function.

Breast milk is a complex and variable biofluid whose composition influences offspring programming [68,69]. Breastfeeding not only has nutritional, immunological, and cognitive benefits, but has also been linked to the prevention of obesity [70]. Breast milk composition reflects maternal nutritional status and dietary intake. Milk vitamins, minerals, and fatty acid composition, for example, are influenced by maternal nutrition [71,72]. Although DHA can be synthesized by the mother from its precursor (α- linolenic acid), DHA obtained from the mother’s diet is a more efficient source of DHA because less than 10% of α- linolenic acid is converted to DHA [73]. Unfortunately, many women, including pregnant women, do not consume the recommended amount of ω-3 and instead consume large amounts of ω-6 fatty acids, increasing the ω-6/ω-3 fatty acid intake ratio [73]. In the present study, we observed that milk production was similar among groups and that milk from MO mothers contained less water, EPA, and DHA and more fat, linoleic acid, and AA. Interestingly, maternal DHA supplementation in obese rats did not modify water, fat, linoleic acid, and EPA concentration; however, DHA was increased while AA was decreased to concentrations similar to C. One rat study found that a dairy fat blend containing as little as 1.5% α-linolenic acid is better than a palm oil blend, even when the recommended DHA and ARA are added. If α-linolenic acid concentrations are increased in the dairy matrix by 50%, there is a further increase in DHA. Dietary conditions can reveal a gender effect in brain PUFA, particularly brain DHA, which was lower in male rats than female rats [74,75]. In humans, it has been reported that when colostrum linoleic acid concentrations were high, lower DHA concentrations were associated with lower IQs. High linoleic dietary intake reduces the biosynthesis of DHA from α-linolenic by competing with the enzymes involved in PUFA metabolism [76]. In the present study, the linoleic acid and DHA milk content were reduced in the MO group.

In humans, DHA accumulation in the brain begins in utero and continues after birth, peaking between the ages of two and four years [77]. The accumulation of fetal LCPUFAs occurs as a result of placental transfer, which is directly dependent on the maternal diet [77,78]. Breastfeeding is recommended for the first six months of life, during which time the infant brain doubles in weight due to an increase in neurons, axons of nerve fibers, and synapses, all of which are rich in DHA [73]. Therefore, an adequate LCPUFA intake during lactation is of particular importance for infant neurological development.

The Food and Drug Administration suggests using body surface area to extrapolate animal doses to human doses [79]. In this study, the control and obese mothers were supplemented with 400 mg/kg of DHA, which according to Reagan-Shaw calculation, is equivalent to 3.9 g/day for a human weighing 60 kg. The dose used in the present study was chosen for three reasons: (1) it corresponds to a fifth of the no observed adverse-effect dose in pregnant rats that is 2000 mg/kg/day [80]; (2) rodents have higher tissue requirements of DHA than humans, since the metabolic rates are higher in rats compared to humans [81]; (3) maternal DHA supplementation during pregnancy at different doses (100, 300, and 500mg/kg) protects rats’ offspring against learning and memory impairment following prenatal exposure to valproic acid [82]. These efficacious doses match well with our dose of 400mg/Kg. The amount of DHA in the milk of DHA-supplemented dams is 0.24 g per 100 g of milk fat, which corresponds to 0.24% of DHA in relation to the total amount of fatty acids; this concentration is much lower than the lowest concentration of DHA (0.32%) contained in the infant formulas used in human studies [83].

Increased consumption of Western diets around the world has resulted in significant changes in individuals’ fatty acid intake, resulting in a much greater ω-6/ω-3 ratio. This rise is well known to have negative health consequences. Control animal studies help to understand mechanisms, especially those that may enable beneficial interventions. In contrast, human observational epidemiological studies only indicate associations. One of the goals of the present study was to obtain firm information that can be used to improve milk composition in obese mothers by providing DHA supplementation. The findings in the present study show that supplementing the diet of obese rat mothers with 400 mg/kg of DHA reduces the ω-6/ω-3 ratio by 33%. This level of reduction is accompanied by beneficial effects on offspring health.

Male and female pups of obese dams do not show differences in milk consumption. However, in MO, pups’ AA milk intake was higher compared with C. EPA milk intake had a tendency to be lower in male (*p* = 0.09) and female (*p* = 0.194) pups from MO, but they were not statistically different. As a result, the ω-6/ω-3 fatty acid intake ratio in the milk intake was higher in MO pups. Maternal DHA supplementation in obese rats reduced pups’ AA, and EPA intake remained similar to MO. The ω-6/ω-3 fatty acid intake ratio showed a clear statistically significant beneficial effect of DHA administration which significantly reduced the ratio, returning the ratio to both MO and C values. In C+DHA milk, DHA concentration and pups’ DHA intake was increased while their ω-6/ω-3 fatty acid intake ratio was reduced. Insufficient ω-3 PUFA intake, as well as an excess of ω-6 PUFA, correlate with various diseases [84], including cancer, cardiovascular, inflammatory, and autoimmune diseases [85]. Milk’s ω-6/ω-3 PUFA ratio influences offspring growth, neurodevelopment, and immunoresponsiveness. In a human study, ω-6 and ω-3 fatty acids during the postnatal period (40 to 44 weeks of gestation) were found to be important factors in early neurodevelopment [86]. In a murine model, it has been reported that increased body weight, fasting insulin and triacylglycerol levels, and higher blood pressure have been linked to the n-6/n-3 PUFA ratio in the perinatal period [87].

DHA is the most abundant PUFA in the brain and has a critical, indispensable role in neuronal membrane function [88]. There is evidence that brain DHA concentrations are decreased in Alzheimer’s disease and that major depression and bipolar disorder have low DHA concentrations and high ω-6/ω-3 fatty acid intake ratios [89]. Therefore, a diet affording an optimal ω-6/ω-3 fatty acid intake ratio may be beneficial for promoting physical and mental wellbeing [90]. EPA has beneficial effects on mood disorders [91].

More detailed evidence on the individual roles of EPA and DHA in brain health will assist appropriate dietary recommendations to improve these neurological conditions.

We and others have published evidence to show that maternal obesity induces offspring cognitive and behavioral alterations [19,92]. In the present study, we observed that both male and female offspring from obese mothers showed high levels of anxiety, as evidenced by a lower number of entries and time spent in the elevated plus maze’s open arms compared to C. However, maternal DHA supplementation prevented the development of this anxious behavior in female but not male rats. This observation falls into the general framework of the investigations showing that many physiological functions and pathological alterations, including those related to the Developmental Origins of Health and Disease area [31,93], are sex-dependent. In the present study, the differences between male and female rats might arise from the establishment of different patterns of connectivity in response to stress. In fact, it has been shown that acute tail-shock stress inhibits estrogen-dependent spine formation in CA1 neurons in female rats while enhancing spine density in male CA1 neurons [94]. Interestingly, spine formation is dependent on testosterone secretion, which is increased by both stress [95,96] and DHA [97].

Several mechanisms, including immune activation, impaired hypothalamic–pituitary–adrenal axis (HPA) activity, and neuroendocrine dysfunction, have been proposed to explain the increased risk of anxiety and depression in obese people [98]. For example, dysregulation of the HPA axis has been suggested to increase the risk of developing stress-related disorders, such as anxiety and depression [99]. Various studies show that increased glucocorticoid concentrations during pregnancy alter HPA axis function and modify offspring behavior. Glucocorticoids easily pass through the blood-brain barrier and affect the limbic system, including, for example, the amygdala, which is a major brain region implicated in the regulation of anxiety-like behavior [25]. Evidence also suggests that the serotonergic system is involved in glucocorticoid-induced HPA programming [100]. Bilateral ventral hippocampal leptin injections in mice affect spatial learning and memory without affecting anxiety-like behavior and locomotor activity in the elevated plus maze and open field [57]. Individuals with mood disorders have been shown to have altered membrane fatty acid composition; in this context, societies with high consumption of fish, which is high in ω-3 PUFAs, appear to have a lower prevalence of major depressive disorders [101]. In addition, new findings suggest that AA status influences depression pathophysiology via effects on serotonin transport [102]. It has been reported that fish oil supplementation in male mice with diet-induced obesity suppresses anxio-depressive behaviors, improves the brain’s anti-inflammatory PUFA lipids, and reduces indices of brain gliosis [98]. According to the authors of the study, increased brain EPA and docosapentaenoic acid and decreased AA from fish oil supplementation contribute to reduced neuroimmune activity and alleviation of diet-induced anxiety. In our model, we found that the MO group had higher AA content in milk as well as higher AA consumption in female and male pups via milk. Maternal supplementation reduced AA milk content in the MO+DHA group, but interestingly, milk AA intake was only reduced in female pups. These findings may partly explain some of the gender differences in anxiety behavior.

In conclusion, we observed that maternal DHA supplementation in obese rats prior to and throughout pregnancy and lactation improves milk composition, reduces the ω-6/ω-3 fatty acid intake ratio, and reduces the offspring anxiety type behavior in female but not in male offspring. Further studies are needed to evaluate the neural mechanisms involved.

## Figures and Tables

**Table 1:** Maternal rat milk composition (ω-3, ω-6, monounsaturated fatty acids-MSA, saturated fatty acids-SFA) at the end of lactation. Control (C), control with DHA (C+DHA), maternal obesity (MO), and maternal obesity with DHA (MO+DHA).

Fatty Acids, %	C	C+DHA	MO	MO+DHA	Interaction
ω-3 PUFAS	0.4 ± 0.01	0.5 ± 0.08	0.4 ± 0.03	0.4 ± 0.03	*p* = n.s.
ω-6 PUFAS	2.8 ± 0.16	2.9 ± 0.39	5.7 ± 0.51 *	4.9 ± 0.29 *	*p* = n.s.
MSA	4.6 ± 0.29	3.5 ± 0.52	6.1 ± 1.42	7.7 ± 0.38 *	*p* = 0.06
SFA	5.8 ± 0.18	5.7 ± 0.44	7.6 ± 0.54 *	7.1 ± 0.34	*p* = n.s.

Mean ± SEM, *n* = 7–8 mothers per group. Two-way multiple analysis of variance (ANOVA) followed by Tukey test. * different vs. their respective control, *p* < 0.05. I = interaction between maternal diet and maternal DHA supplementation, n.s. = not significant.

**Table 2:** Pups’ milk intake (ω-3, ω-6, monounsaturated fatty acids-MSA, saturated fatty acids-SFA) at the end of lactation. Control (C), control with DHA (C+DHA), maternal obesity (MO), and maternal obesity with DHA (MO+DHA).

Fatty acids, mg/h	C	C+DHA	MO	MO+DHA	Interaction
Male					
ω-3 PUFAS	3 ± 0.4	4 ± 1	4 ± 0.9	4 ± 1	*p =* n.s.
ω-6 PUFAS	20 ± 3	20 ± 6	60 ± 10 *	40 ± 10	*p =* n.s.
ω-6/ ω-3 ratio	7.4 ± 0.36 ^a^	5.7 ± 0.17 ^b^	15.8 ± 0.42 ^a^*	11.2 ± 0.54 ^b^*	*p* < 0.05
MSA	36 ± 6	29 ± 9	68 ± 29	64 ± 23	*p* = n.s.
SFA	40 ± 6	50 ± 10	80 ± 20	60 ± 20	*p* = n.s.
Female					
ω-3 PUFAS	2 ± 0.3 ^a^	5 ± 0.8 ^b^	3 ± 0.7	3 ± 0.7	*p* < 0.05
ω-6 PUFAS	10 ± 2	30 ± 4	60 ± 10 *	40 ± 7	*p* < 0.05
ω-6/ω-3 ratio	7.4 ± 0.36 ^a^	5.7 ± 0.17 ^b^	15.8 ± 0.42 ^a^*	11.2 ± 0.54 ^b^*	*p* < 0.05
MSA	24 ± 4	36 ± 8	58 ± 23	56 ± 12	*p* = n.s.
SFA	30 ± 4	50 ± 6	70 ± 20*	50 ± 10	*p* < 0.05

Mean ± SEM, *n* = 7–8 rats from different litters. Two-way multiple analysis of variance (ANOVA) followed by Tukey test. Within the same group, means labeled with different letters differ, *p* < 0.05. * different vs. their respective control, *p* < 0.05. Interaction between maternal diet and maternal DHA supplementation, n.s. = not significant.

**Table 3:** Open field test in male and female offspring at PND 130. Control (C), control with DHA (C+DHA), maternal obesity (MO), and maternal obesity with DHA (MO+DHA).

	C	C+DHA	MO	MO+DHA	Interaction
**Male**					
Total distance, m	47.1 ± 1.6	46.1 ± 3.5	44.9 ± 6.9	56.1 ± 9.8	*p* = n.s.
Center zone entries, #	10.2 ± 1.7	8.8 ± 1.1	3.0 ± 0.4 *	7.7± 1.9	*p* = 0.06
Center zone time, s	13.2 ± 1.6	13.0 ± 2.3	4.3 ± 1.5	12.9 ± 5.3	*p* = n.s.
Center zone distance, m	2.9 ± 0.6	2.2 ± 0.3	0.87 ± 0.3 *	2.0 ± 0.4	*p* = 0.06
**Female**					
Total distance, m	57.8 ± 3.8	62.3 ± 2.7	49.2 ± 3.5	61.5 ± 4.9	*p* = n.s.
Center zone entries, #	13.0 ± 2.9	13.7 ± 2.1	7.7 ± 1.9	12.6 ± 2.5	*p* = n.s.
Center zone time, s	21.1 ± 5.0	22.6 ± 5.1	15.9 ± 4.7	20.7 ± 4.5	*p* = n.s.
Center zone distance, m	4.1 ± 0.9	3.7 ± 0.7	2.3 ± 0.6	3.8 ± 0.5	*p* = n.s

Mean ± SEM, *n* = 7–8 rats from different litters. Two-way multiple analysis of variance (ANOVA) followed by Tukey test, * different vs. their respective control, *p* < 0.05. n.s. not significant. Interaction between maternal diet and maternal DHA supplementation, # = number, n.s. = not significant.

## Data Availability

The data supporting the research for this study is available within the manuscript.

## Abbreviations

Arachidonic acid (AA), control group (C), control group + DHA supplementation (C+DHA), days of lactation (dL), docosahexaenoic acid (DHA), eicosapentaenoic (EPA), elevated plus maze (EPM), hypothalamic–pituitary–adrenal axis (HPA), long-chain polyunsaturated fatty acids (LCPUFA), maternal obesity group (MO), maternal obesity group + DHA supplementation (MO+DHA), monounsaturated fatty acids (MSA), open field (OF), postnatal day (PND), saturated fatty acids (SFA).
